# A novel Laccase Biosensor based on Laccase immobilized Graphene-Cellulose Microfiber Composite modified Screen-Printed Carbon Electrode for Sensitive Determination of Catechol

**DOI:** 10.1038/srep41214

**Published:** 2017-01-24

**Authors:** Selvakumar Palanisamy, Sayee Kannan Ramaraj, Shen-Ming Chen, Thomas C. K. Yang, Pan Yi-Fan, Tse-Wei Chen, Vijayalakshmi Velusamy, Sonadevi Selvam

**Affiliations:** 1 Electroanalysis and Bioelectrochemistry Lab, Department of Chemical Engineering and Biotechnology, National Taipei University of Technology, Taipei City, Taiwan, ROC; 2 PG & Research department of Chemistry, Thiagarajar College, Madurai-09, Tamilnadu, India; 3 Department of Chemical Engineering and Biotechnology, National Taipei University of Technology, No. 1, Section 3, Chung-Hsiao East Road, Taipei City, Taiwan; 4 Division of Electrical and Electronic Engineering, School of Engineering, Manchester Metropolitan University, Manchester, M1 5GD, United Kingdom

## Abstract

In the present work, we demonstrate the fabrication of laccase biosensor to detect the catechol (CC) using laccase immobilized on graphene-cellulose microfibers (GR-CMF) composite modified screen printed carbon electrode (SPCE). The direct electrochemical behavior of laccase was investigated using laccase immobilized different modified SPCEs, such as GR/SPCE, CMF/SPCE and GR-CMF/SPCE. Compared with laccase immobilized GR and CMF modified SPCEs, a well-defined redox couple of Cu^I^/Cu^II^ for laccase was observed at laccase immobilized GR-CMF composite modified SPCE. Cyclic voltammetry results show that the as-prepared biosensor has 7 folds higher catalytic activity with lower oxidation potential towards CC than SPCE modified with GR-CMF composite. Under optimized conditions, amperometric *i-t* method was used for the quantification of CC, and the amperometric response of the biosensor was linear over the concertation of CC ranging from 0.2 to 209.7 μM. The sensitivity, response time and the detection limit of the biosensor for CC is 0.932 μMμA^−1^ cm^−2^, 2 s and 0.085 μM, respectively. The biosensor has high selectivity towards CC in the presence of potentially active biomolecules and phenolic compounds. The biosensor also accessed for the detection of CC in different water samples and shows good practicality with an appropriate repea.

Over the past two decades, a sensitive and real time detection of phenolic compounds has received substantial interest to the scientific community owing to their high toxicity on the environment, eco system and human health^1^. Moreover, the phenolic compounds are highly toxic organics, has been widely used in different industrial products and can easily accumulate in the environment and eco system^2^,^3^. Among different phenolic compounds, catechol (CC) is an ortho isomer of benzenediols, has classified as a periodic toxic pollutant by the US Environmental Protection Agency and the European Union due to its poor biodegradability and high toxicity on human health and eco system^4^,^5^. Therefore, a simple and real time detection of trace levels of CC in environmental samples is of great importance. To date, different analytical methods have been successfully used for the detection of CC such as high performance liquid chromatography^6^, flow-injection analysis^7^, chemiluminescence^8^, gas chromatography-mass spectrometry^9^ and electrochemical methods^10^. The traditional chromatographic methods are highly sensitive towards CC, yet they are required sample pretreatment, expensive, not portable and often time consuming when compared with electrochemical methods^10^. Till date, different nano and micromaterials have been utilized in electrochemical methods for the sensitive detection of CC, such as carbon nanomaterials, metal and metal alloy nanoparticles, metal oxides, conducting polymers and so on^11^,^12^,^13^,^14^. In addition, different electrode modifications or pretreatments have been realized for the sensitive quantification of CC^15^,^16^. However, the selective detection of CC is still challenging in the presence of isomers of benzenediols.

Recently, the fabrication of enzyme based biosensors has received much attention for selective and sensitive detection of CC, since the enzyme based biosensors are highly sensitive and selective towards CC than non-enzymatic CC sensors^17^. For instance, tyrosinase, polyphenol oxidase and laccase based biosensors have been widely used for selective detection of polyphenolic compounds including CC^18^. Among them, laccase is a blue multi-copper-oxidase and the largest subgroup of multicopper oxidases, has more specific advantages such as the ability to catalyze electron-transfer reactions and high stability over tyrosinase and polyphenol oxidase based biosensors^18^. Different nano and micromaterials modified electrodes have been used for immobilization of laccase, since direct immobilization of laccase on unmodified electrode is difficult^18^. For instance, carbon nanomaterials^19^, metal nanoparticles^20^,^21^, metal oxides^21^, conducting polymers^22^, and ionic liquids^23^ have been used as an immobilization matrix for laccase. Graphene (GR) is a 2D carbon nanomaterial, has showed an extraordinary thermal and electrical properties than other carbon nanoforms such as fullerene and carbon nanotubes^24^,^25^. Recently, GR has been widely used as a support for fabrication of biosensors due to its high conductivity and biocompatibility^26^,^27^. However, the direct immobilization of redox active enzymes on GR surface is difficult due to its strong hydrophobic nature and the presence of only sp^2^ hybridized carbon atoms^28^. Hence, the GR based composites have been largely used for the immobilization of laccase^29^,^30^,^31^. It is reported earlier that the carbohydrate polymers and surfactants dispersed GR has been used as a potential material for immobilization of range of redox active proteins including laccase^31^. In addition, carbohydrate polymers have highly enriched with hydrophilic chemical groups on GR and result into the formation of water soluble GR hybrids^32^. Among different carbohydrate polymers, cellulose microfibers (CMF) are hydrophilic and water-insoluble carbohydrate polymer, has been served as a promising biomaterial for immobilization of redox active proteins owing to its unique chemical properties and high biocompatibility^33^. In addition, CMF has showed a high surface area and high porosity and has a tendency to bind with the range of conductive materials including carbon nanomaterials^33^,^34^,^35^,^36^. Despite the unique chemical properties of CMF, in the present work we have used CMF as a dispersing agent for GR and the resulting GR-CMF composite is used as an immobilization matrix for laccase. Moreover, the induced hydrophilic nature of CMFs could effectively prevent aggregation of GR and forms the stable GR-CMF composite for immobilization of laccase. According to our literature review, all the reported GR/cellulose composites have been prepared by the chemical reduction of graphene oxide with cellulose^34^,^35^,^36^,^37^, yet no attempt has been made so far for the direct preparation of GR-CMF composite. In addition, the direct preparation of GR-CMF does not involve any toxic chemical regents when compared to chemically prepared GR-CMF composites. To the best of our knowledge, the GR-CMF based composites have never been demonstrated yet for any electrochemical biosensor applications including immobilization matrix for any redox active enzymes or proteins.

In the present work, a highly sensitive and selective CC biosensor was developed based on laccase immobilized GR-CMF composite modified screen printed carbon electrode (SPCE). The bioelectrochemical redox behavior of laccase was investigated in different laccase immobilized different modified SPCEs. The schematic representation for the fabrication of the laccase biosensor is shown in Fig. 1. The resulting biosensor was further used for the detection of CC and was quantified using an amperometric method.

## Results and Discussion

### Characterizations of the as-prepared materials

**Figure 2:**
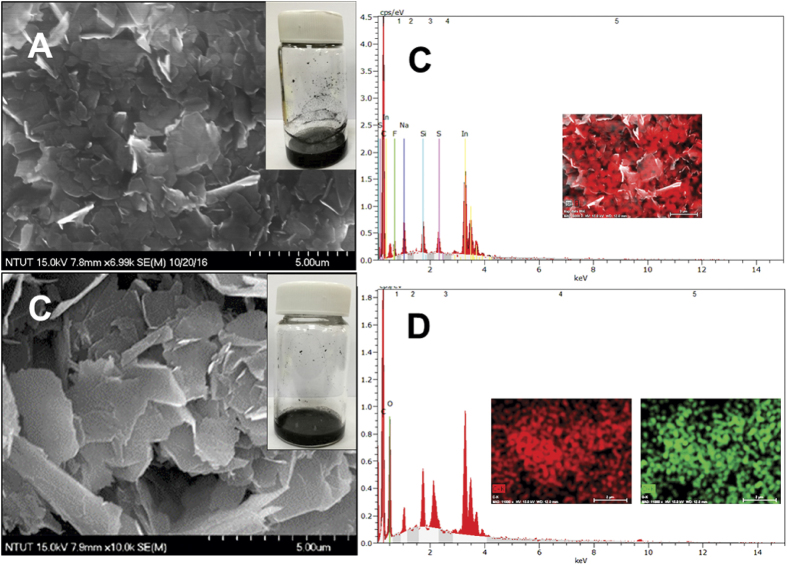
High resolution SEM images of GR (**A**) and GR-CMF composite (**C**). Optical images of GR dispersion in DMF (inset of A) and CMF (inset of C). The EDS and elemental mapping of GR (**B**) and GR-CMF composite (**D**).

Figure 2 displays the high resolution SEM images of GR (A) and GR-CMF composite (C). Figure 2C and D shows the corresponding EDS and elemental mapping of GR and GR-CMF composite. The SEM image of pristine GR is appeared as a few layers nanoflakes and its EDS and elemental mapping is confirmed the presence of carbon, which suggests the pure nature of GR flakes (A). On the other hand, the SEM image of GR-CMF composite shows that a typical 3D morphology and the GR nanoflakes were well exploited when dispersed in CMF (B).

**Figure 3:**
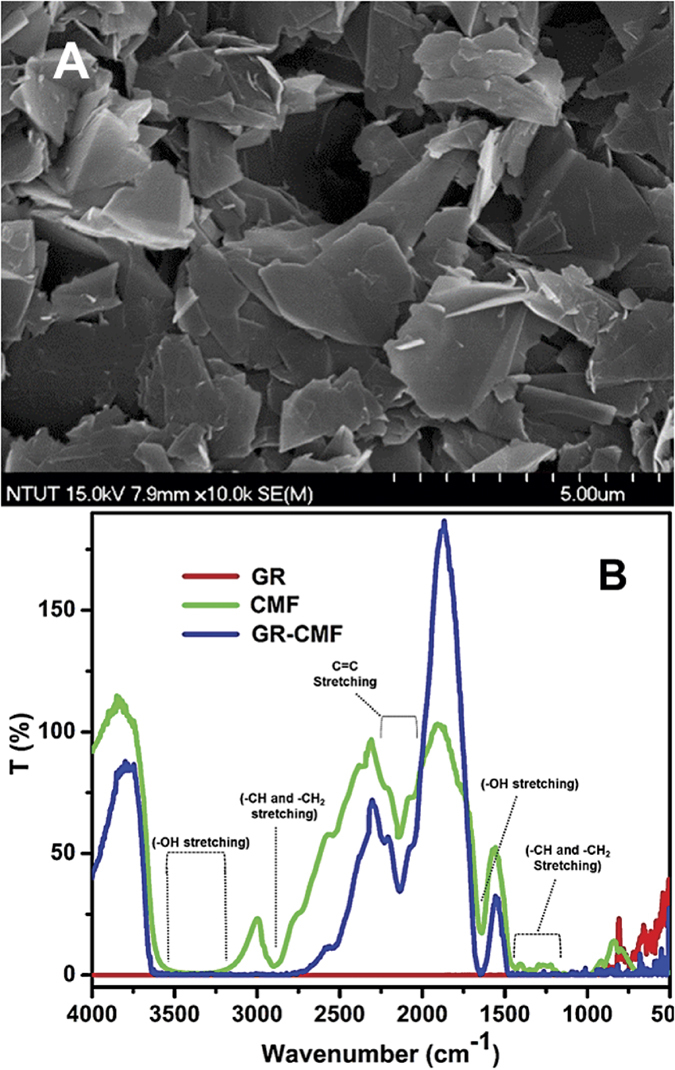
(**A**) High resolution SEM image of SPCE modified GR-CMF composite. (**B**) FTIR spectra of CMF (green line), GR (red line) and GR-CMF composite (blue line).

In addition, the surface of the GR was smother in GR-CMF when compared to pristine GR, which is due to the presence of CMF. The digital photographs of GR-CMF composite (Fig. 2C inset) is also confirmed that CMF is more suitable dispersing agent for GR. It is also noted that the obtained GR-CMF dispersion is more consistent with the GR dispersed in dimethylformamide (DMF). Furthermore, the EDS and elemental mapping of the GR-CMF composite (Fig. 2D) confirmed the presence of carbon and oxygen which are resulting from the presence of GR and CMF. In addition, the surface morphology of GR-CMF composite did not change upon coated on the SPCE surface, as shown in Fig. 3A. The above results confirmed the successful formation of GR-CMF composite.

FTIR spectroscopy is further used to confirm the presence of CMF on GR-CMF composite. Figure 3B shows the typical FTIR spectra of CMF (green line), GR (red line) and GR-CMF composite (blue line). The FTIR spectrum of GR (red line) is found featureless in the finger print region, while the FTIR spectrum of GR-CMF composite (blue line) shows a broad characteristic vibration band at 3300–3500 cm^−1^, is corresponding to stretching vibrations of –OH group^38^. In addition, two additional bands were observed at 2892 and 2220 cm^−1^, which are due to stretching of –CH and –CH_2_ from CMF [**38**]. A sharp characteristic band is appeared at 1640 cm^−1^, is due to the vibrations of –OH from absorbed water of CMF^38^. The similar characteristic bands were observed for the FTIR spectrum CMF (green line). The results confirmed that presence of CMF in GR-CMF composite. Raman spectroscopy has been used to confirm the purity of utilized GR in GR-CMF composite, since it is an ideal technique to characterize carbon nanostructures. The intensity ratio (I_2D_/I_G_) for 2D and G band ratio of GR-CMF was calculated as 0.99 (figure not shown), which confirms that the utilized GR in the GR-CMF composite is few layers GR^39^.

### Direct electrochemistry of laccase on different modified SPCEs

**Figure 4:**
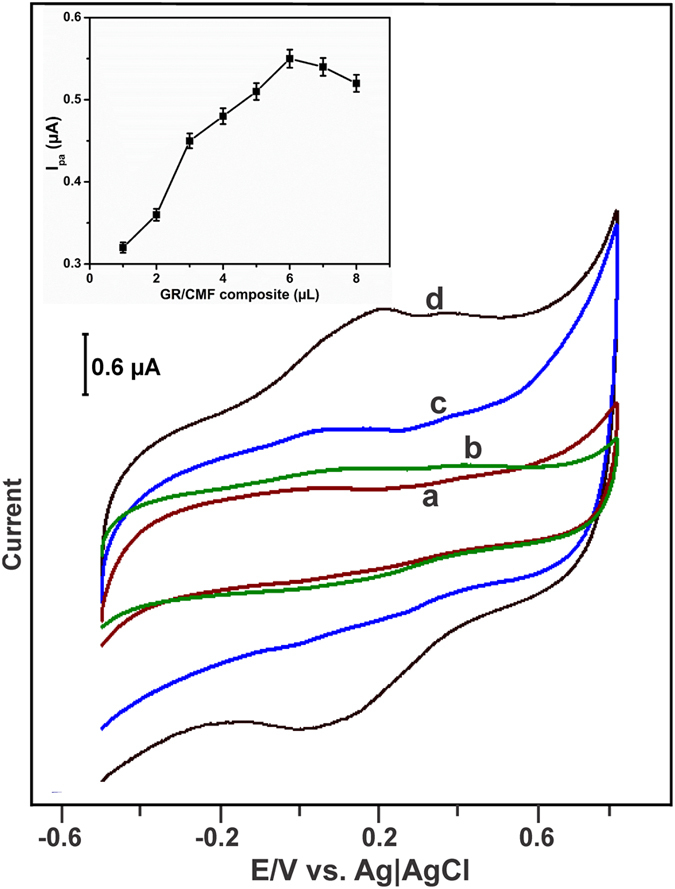
Cyclic voltammetry response of laccase immobilized on bare SPCE (**a**), CMF/SPCE (**b**), GR/SPCE (**c**) and GR-CMF/SPCE (**d**) in pH 5.0 at a scan rate of 100 mV/s. Inset shows the effect of different drop coated amount of GR/CMF composite vs. anodic redox peak current response of immobilized laccase on GR/CMF composite.

In order to study the direct electrochemistry of laccase, the laccase was immobilized on different modified electrodes and its electrochemical redox behavior was studied using cyclic voltammetry. Since laccase contains Cu^I^/Cu^II^ (T2/T3 cluster) as a redox active center and is responsible for direct bioelectrochemical behavior of laccase^40^. As shown in Fig. 4 inset, a maximum anodic redox peak current of laccase was found for laccase immobilized on 6 μL drop coated GR/CMF composite than laccase immobilized 5 and 7 μL drop coated composite modified electrode. Hence, it is used as an optimum for further electrochemical studies.

Figure 4 shows the cyclic voltammetry response of laccase immobilized bare SPCE (a), CMF/SPCE (b), GR/SPCE (c) and GR-CMF/SPCE (d) in pH 5.0 at a scan rate of 100 mV/s. The cyclic voltammetry measurements were carried out in N_2_ atmosphere in the potential range between −0.5 to +0.7 V. The laccase immobilized unmodified SPCE did not show any obvious redox couple for laccase, which indicates that the unmodified SPCE is not suitable for immobilization of laccase. While, a weak redox couple is appeared for laccase immobilized CMF/SPCE, which indicates that CMF provide a suitable matrix for immobilization of laccase than bare SPCE. On the other hand, laccase immobilized GR/SPCE shows only an anodic peak at +0.103 V and the reversible cathodic peak of laccase is absent. The result indicates that the direct electrochemistry of laccase is not favorable on GR and unmodified SPCEs. However, a well-defined redox couple was appeared for laccase at laccase immobilized GR-CMF/SPCE and the anodic and cathodic peak potentials were observed at +0.212 and +0.065 V, which are due to the T2/T3 cluster of Cu^I^/Cu^II^ redox active center of laccase^40^. In addition, the direct electrochemical redox behavior of laccase was 6 folds enhanced at laccase immobilized GR-CMF/SPCE when compared with laccase immobilized CMF/SPCE. The good biocompatibility of CMF is providing a suitable matrix for the orientation of more number of laccase and the high surface area of GR provides an enhanced redox intensities of laccase on GR-CMF modified SPCE.

**Figure 5:**
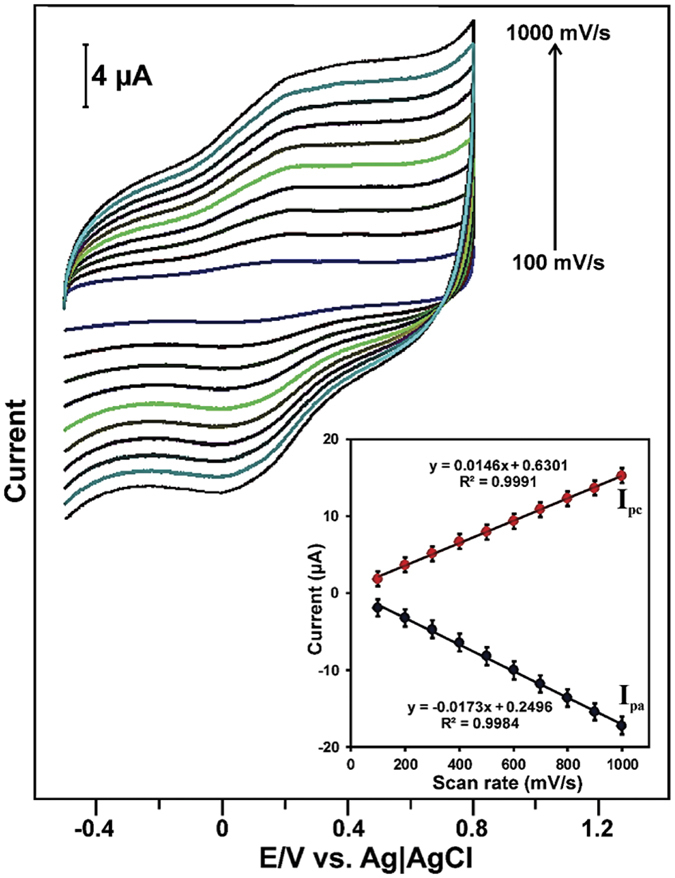
Cyclic voltammetry response obtained for laccase immobilized on GR-CMF modified SPCE in pH 5.0 at different scan rates from 100 to 1000 mV/s. Inset is the linear plot for anodic and cathodic peak current of laccase redox couple vs. scan rate.

The effect of scan rate on the electrochemical redox behavior of laccase was studied using laccase immobilized GR-CMF modified SPCE by cyclic voltammetry. Figure 5 shows the cyclic voltammograms of laccase immobilized GR-CMF modified SPCE in pH 5.0 for the different scan rates (100 to 1000 mV/s). It can be seen that the anodic and cathodic peak current of laccase redox couple increases with increasing the scan rate from 100 to 1000 mV/s. Furthermore, the anodic and cathodic peak potentials were found unchanged upon increasing the scan rates from 100 to 1000 mV/s.

As shown in Fig. 5 inset, the scan rate vs. anodic and cathodic peak current of laccase redox couple was found linear and the linear regression equations were expressed as: I_pa_ (μA) = −0.0173 + 0.2496 mV/s (R^2^ = 0.9984) and I_pc_ (μA) = −0.0146 + 0.6301 mV/s (R^2^ = 0.9991). The result further indicates that direct electrochemistry of laccase redox couple was surface controlled electrochemical process on laccase immobilized GR-CMF composite modified electrode.

**Figure 6:**
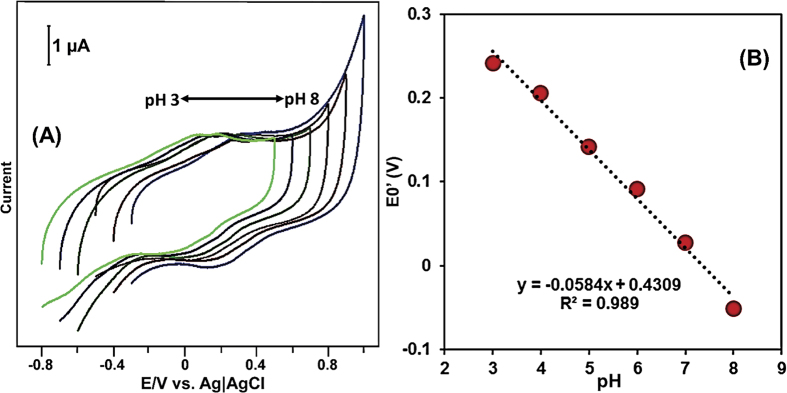
(**A**) Cyclic voltammetric behavior of GR-CMF/laccase modified SPCE in different pH (pH 3–8) at a scan rate of 100 mV/s. (**B**) Linear plot for formal potential of laccase redox couple vs. pH.

The direct bioelectrochemical behavior of laccase was studied in different pH using cyclic voltammetry, since it is well-known that the redox electrochemical behavior of laccase is pH dependent. Figure 6A displays the cyclic voltammetry response of laccase immobilized GR-CMF composite modified SPCE in different pH at a scan rate of 100 mV/s. It can be clearly seen that the anodic and cathodic peak potential of laccase redox couple has highly affected by pH and the formal potential (E^0^′, where E^0^ = (E_pa_ + E_pc_/2) of laccase had a linear dependence with wide pH (pH 3–8), as shown in Fig. 6B. The linear regression equation was found as E^0^′ = −0.0584 + 0.4309 V/pH with the correlation coefficient of 0.989. The obtained slope value (−58.4 mV/pH) is clearly indicates that the redox electrochemical behavior of laccase is involving of an equal number of electrons and protons. In addition, the obtained slope value is in good agreement with the reported theoretical value of the Nernstian equation for an equal number of electrons and protons involved reversible electrochemical process, as reported elsewhere^41^.

### Electrocatalytic oxidation of CC

**Figure 7:**
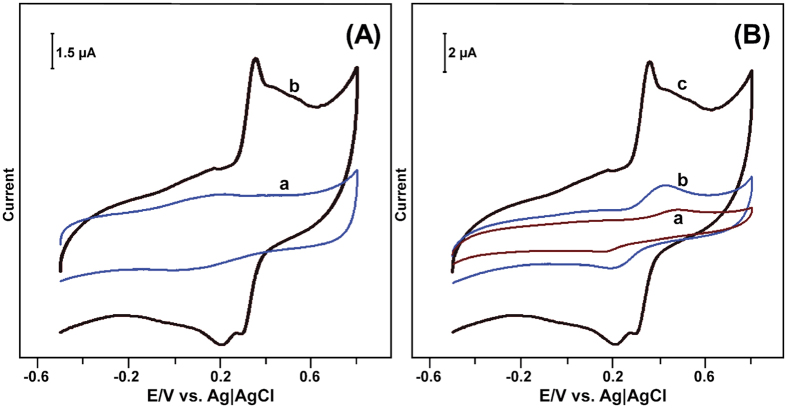
(**A**) Cyclic voltammetric response obtained for GR-CMF/laccase modified SPCE in the absence (a) and presence (b) of 50 μM CC in pH 5 at a scan rate of 100 mV/s. (**B**) At similar conditions, the cyclic voltammetry response of CMF (a), GR-CMF (b) and GR-CMF/laccase (c) modified SPCEs towards 50 μM CC.

To verify the electrocatalytic ability of the biosensor, the laccase immobilized GR-CMF composite modified SPCE was used for the oxidation of CC by cyclic voltammetry. Figure 7A shows the cyclic voltammetry response of the laccase immobilized GR-CMF composite modified SPCE in the absence (a) and presence (b) of 50 μM CC in pH 5.0 at a scan rate of 100 mV/s. In the absence of CC, a well-defined redox couple of laccase was observed at +0.212 and +0.065 V. Whereas a sharp oxidation peak was appeared at +0.404 V in the presence of 50 μM CC, which is due to the oxidation of CC to 1,2-benzoquinone by laccase redox couple.

**Figure 8:**
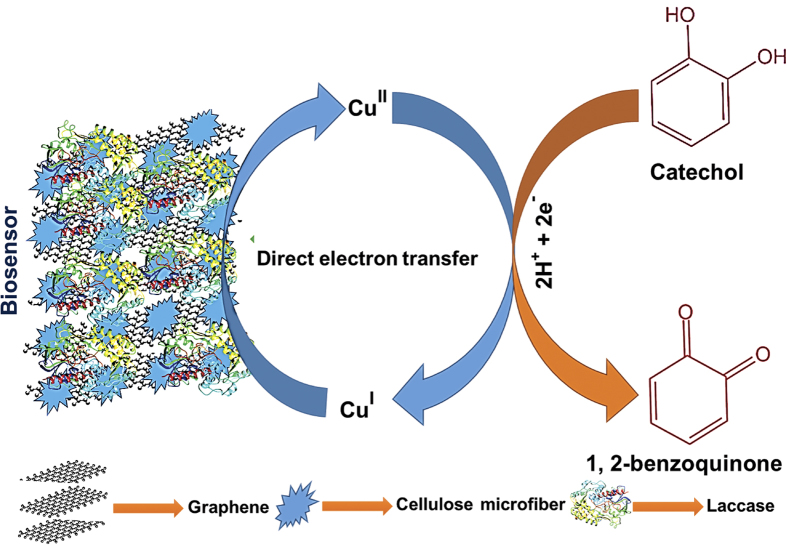
Schematic representation for the electrochemical redox behavior of laccase and electro-oxidation mechanism of CC by the as-prepared laccase biosensor.

We have also compared the electrocatalytic activity of the as-prepared biosensor with the CMF and GR-CMF modified SPCEs towards the detection of 50 μM CC. Figure 7B shows the cyclic voltammetry response of CMF (a), GR-CMF (b) and GR-CMF/laccase (c) modified SPCEs in 50 μM CC containing pH 5.0 at a scan rate of 100 mV/s. It can be seen that the GR-CMF composite modified SPCE shows a weak redox couple for CC and the oxidation peak of CC was appeared at +0.434 V. On the other hand, the oxidation peak of CC is appeared at +0.486 V at CMF modified SPCE and the observed oxidation peak current of CC was lower than those observed at GR-CMF modified electrode. However, the laccase immobilized GR-CMF composite modified SPCE shows 5.5 and 7.0 folds enhanced oxidation peak current response to CC than GR-CMF and CMF modified SPCEs. Moreover, the observed oxidation peak potential of CC at the biosensor was 30 and 82 mV lower than those observed at GR-CMF and CMF modified SPCEs. The enhanced electrocatalytic activity and lower oxidation potential of the biosensor towards CC is due to the presence of Cu^I^/Cu^II^ redox couple of laccase on the biosensor. The above results clearly indicate that laccase immobilized GR-CMF composite modified SPCE has high catalytic activity and lower oxidation potential for the detection of CC than other modified SPCEs. The plausible electrochemical oxidation mechanism of CC by laccase redox couple (Cu^I^/Cu^II^) of the biosensor is shown in Fig. 8.

### Amperometric determination of CC

**Figure 9:**
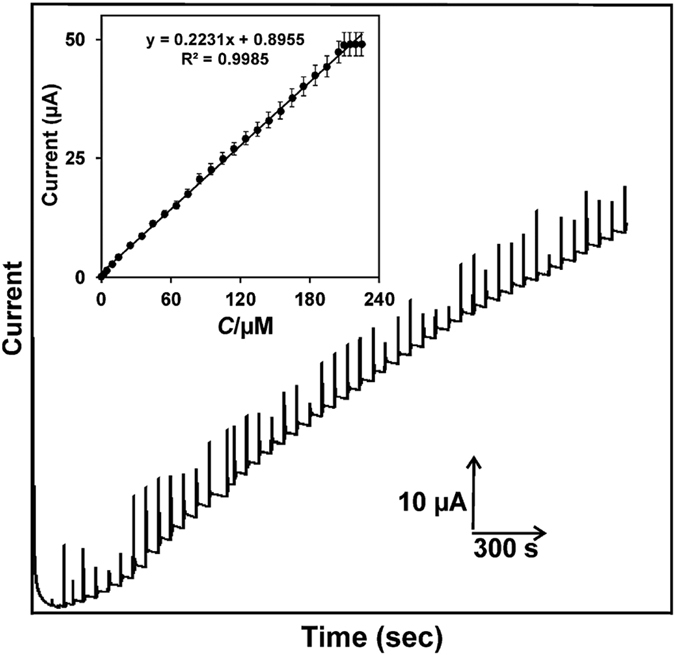
Amperometric *i-t* response of the GR-CMF/laccase modified SPCE for different concentration additions (0.2 to 224.7 μM) of CC into the constantly stirred 0.1 M sodium phosphate buffer pH 5.0; Working potential = +0.4 V. Inset is the linear plot for the amperometric current response vs. [CC].

Amperometric *i-t* method was used for the determination of CC using as-prepared laccase immobilized GR-CMF modified SPCE. Amperometric *i-t* measurements were performed in constantly stirred N_2_ saturated 0.1 M sodium phosphate buffer pH 5.0 with an electrode working potential of +0.4 V (selected from CV results). Under optimized conditions, the amperometric *i-t* response of different concertation additions of CC (0.2 to 224.7 μM) was measured using the as-prepared laccase biosensor, and the obtained amperometric results are displayed in Fig. 9. It can be clearly seen that the biosensor exhibited a stable and well-defined amperometric current response for the addition of different concertation of CC into the constantly stirred N_2_ saturated pH 5.0. The biosensor shows a stable and well-defined amperometric response towards CC from 0.2 to 224.7 μM.

The response time of biosensor towards CC was calculated as 2 s, which indicates the fast electrocatalytic oxidation of CC. Furthermore, the amperometric response of the biosensor was linear over the CC concentrations ranging from 0.2 to 209.7 μM with the correlation coefficient of 0.9985 (Fig. 9 inset). The limit of detection (LOD) of the biosensor was estimated as 0.085 μM using the IUPAC recommendations (S/N = 3). The sensitivity (sensitivity = slope/ESCA, where slope = 0.2231 and ESCA = 0.25 cm^2^) of the biosensor was calculated as 0.932 μAμM^−1^ cm^−2^. In order to evaluate the novelty and superiority of the biosensor, we made the comparison table for analytical performance of the as-prepared biosensor with previously reported laccase biosensors for CC and the comparative results are shown in Table 1. The comparative results clearly show that the as-prepared laccase biosensor exhibited a lower LOD (85 nM) towards CC than previously reported CC biosensors based on laccase immobilized on reduced graphene oxide supported palladium–copper alloyed nanocages^42^, nitrogen-doped ordered mesoporous/PVA matrix^43^, copper-containing ordered mesoporous carbon/chitosan matrix^44^, electrospun copper/carbon composite nanofibers^45^, 1-aminopyrene functionalized reduced graphene oxide^46^, carbon nanotubes–chitosan composite^47^, polyaniline^48^, multi-walled carbon nanotubes^49^ and ZnO sol-gel/chitosan modified electrodes^50^. However, the LOD of the as-fabricated CC biosensor is higher than the LOD (76 nM) of previously reported CC biosensor based on laccase immobilized reduced graphene oxide–glycol chitosan nanohybrid modified electrode, yet the sensitivity and linear response range of our biosensor is more comparable for the determination of CC. In addition, the linear response range and sensitivity of our biosensor is comparable with the previously reported CC biosensors, as shown in Table 1. Therefore, the laccase immobilized GR-CMF modified SPCE can be used as a sensitive probe for low level detection of CC in lab and environmental samples.

### Selective detection of CC using as-prepared laccase biosensor

The selectivity of the biosensor is more important in the presence of laccase subtracts such as polyphenolic and aminophenol compounds. Hence, the selectivity of the as-prepared laccase biosensor toward detection of CC was evaluated in the presence of mono, di, polyphenol and aminophenol compounds.

**Figure 10:**
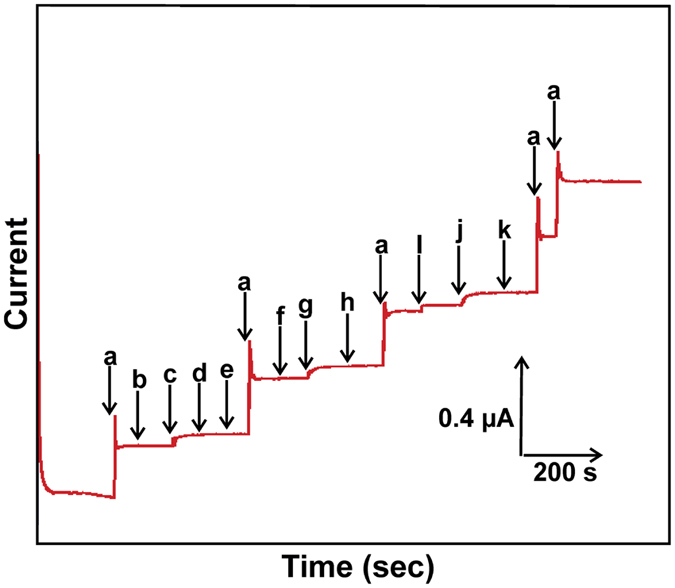
Amperometric *i-t* response of the biosensor for the addition of 1 μM CC (a) and 50 μM additions of hydroquinone (b), dopamine (c), resorcinol (d), phenol (e), 2-amino phenol (f), pyrogallol (g), caffeic acid (h), gallic acid (i), catechin (j) and phloroglucinol (k) into the constantly stirred 0.1 M sodium phosphate buffer pH 5.0.

Figure 10 shows the amperometric i-t response of the biosensor for the addition of 1 μM CC (a) and 50 μM additions of hydroquinone (b), dopamine (c), resorcinol (d), phenol (e), 2-amino phenol (f), pyrogallol (g), caffeic acid (h), gallic acid (i), catechin (j) and phloroglucinol (k) into the constantly stirred 0.1 M sodium phosphate buffer pH 5.0 with an operational working potential of +0.4 V. It can be seen that the biosensor showed a stable and well-defined amperometric *i-t* response for the addition of 1 μM CC. On the other hand, 50 μM additions of hydroquinone, resorcinol, phenol, 2-amino phenol, caffeic acid, and phloroglucinol did not show any discernible current response on laccase immobilized GR-CMF modified SPCE. On the other hand, 50 μM additions of dopamine, pyrogallol, gallic acid and catechin shows a weak response on the biosensor modified SPCE, while the observed response current of these laccase substrates is lower than the current response observed for CC. It is well known that laccase have shown excellent electrocatalytic activity toward mono, di, polyphenols and aminophenols by the reduced T1 active site to T2 and T3 copper sites^18^. However, above given the point the as-prepared laccase biosensor activity is more favorable to CC than other polyphenolic and aminophenol compounds. The results confirmed that the as-prepared laccase biosensor can be used for the selective detection of CC in the presence of potentially active laccase subtracts such as polyphenolic and aminophenol compounds.

### Storage stability and practicality of the laccase biosensor

**Figure 11:**
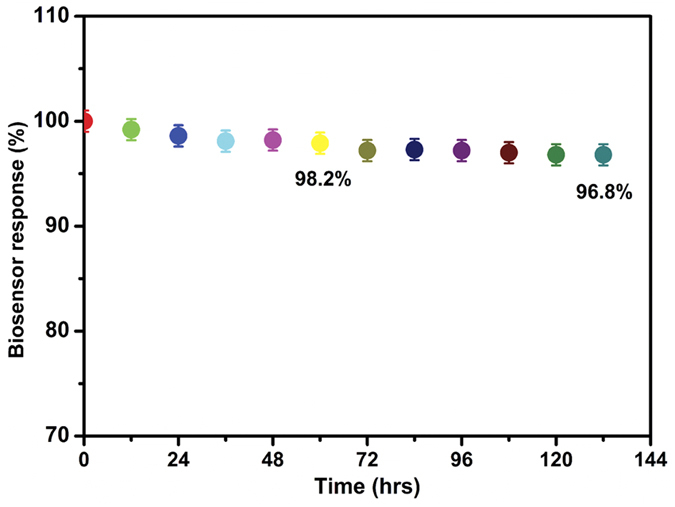
The storage stability of the GR-CMF/laccase modified SPCE for detection of 50 μM CC in 0.1 M sodium phosphate buffer pH 5.0.

The storage stability of the biosensor towards the detection of 50 μM CC was investigated by cyclic voltammetry and the results are shown in Fig. 11. The experimental conditions are similar as of in Fig. 7A. The oxidation peak current response of 50 μM CC was investigated periodically (every 12 h) using laccase immobilized GR-CMF modified SPCE. It can be seen from Fig. 11, the biosensor retains 98.6, 98.2, 97.2 and 96.8% its initial oxidation peak current response to CC after the 24, 60, 96 and 132 h storage in 0.1 M sodium phosphate buffer pH 5.0. The result reveals that the as-prepared laccase biosensor has excellent high storage stability towards CC.

The accuracy of the as-prepared laccase biosensor was also further investigated using cyclic voltammetry and the experimental conditions are similar to Fig. 7A. Five independently prepared laccase immobilized GR-CMF modified SPCEs were used for the detection of 50 μM CC, and gives the relative standard deviation (RSD) about 2.6%. The observed RSD value of the biosensor indicates that the as-prepared biosensor has high accuracy towards the detection of CC.

The practical applicability of the biosensor is more important in order to use for the real sample analysis. Hence, we have tested the practical applicability of the laccase immobilized GR-CMF modified SPCE in CC containing different water samples (tap and river water). Amperometric *i-t* method was used for the real sample analysis and the experimental conditions are similar to Fig. 9. The real samples were tested and are CC free, then the known concertation of CC containing tap and river water was used for real sample analysis. The pH of the water samples was adjusted to pH 5.0 before the real sample analysis. The standard addition method was used for the calculation of recovery of CC and obtained the recoveries of CC were summarized in Table 2. The Table 2 clearly reveals that the biosensor has satisfactory recovery towards CC and the recoveries were 98.8, and 96.4% in tap and river water samples with an acceptable RSD. The above results clearly indicate that laccase immobilized GR-CMF modified SPCE can be used for real-time detection of CC in the environmental samples.

## Conclusions

In conclusion, a sensitive and selective laccase biosensor has been developed for the detection of CC using laccase immobilized GR-CMF modified SPCE for the first time. The direct electrochemical behavior of laccase has been investigated on laccase immobilized different modified SPCEs. The cyclic voltammetry results revealed that the laccase immobilized GR-CMF modified SPCE has enhanced direct electrochemical behavior for laccase than laccase immobilized other modified SPCEs. The high conductivity of GR and good biocompatibility of CMF are result into the firm attachment of laccase on the composite modified SPCE. The as-prepared biosensor exhibited a low LOD (85 nM), high sensitivity (0.932 μAμM^−1^ cm^−2^), fast response (2 s) with a wider linear response range (up to 209.7 μM) for the detection of CC. The biosensor also had good reproducibility and long term storage stability for the detection of CC. The good recovery of CC in different water samples revealed that the as-fabricated laccase biosensor has great potential for monitoring of CC in the investigated water samples and environmental samples. As a future perspective, we strongly believed that the as-prepared GR-CMF composite can be used as an immobilization matrix for other redox active proteins in the future.

## Experimental

### Material and Methods

Catechol and cellulose microfibers (medium) powder were purchased from Sigma Aldrich. 8 nm graphene nanoflakes was purchased from UniRegion Bio-Tech, Taiwan. Laccase from Trametes versicolor mushroom was obtained from Sigma Aldrich and used as received. All other chemicals used in this study were purchased from Sigma Aldrich. Screen printed carbon electrodes (geometric area = 0.08 cm^2^) were purchased from Zensor R&D, Taiwan. 0.1 M sodium phosphate buffer, pH 5.0 was used as the supporting electrolyte, and was prepared by using 0.1 M Na_2_HPO_4_ and NaH_2_PO_4_ in doubly distilled water and the pH was adjusted with 0.1 M H_2_SO_4_ and 0.1 M NaOH.

Hitachi S-4300SE/N High Resolution Schottky Analytical VP scanning electron microscope (SEM) was used for the surface characterizations of the as-prepared materials. Hitachi S-4300SE/N High Resolution Schottky Analytical VP SEM attached BRUKER AXS elemental analyzer was used for the elemental analysis (EDS) and elemental mapping of the composite. JASCO FTIR-6600 spectrometer was used for the Fourier transform infrared (FTIR) spectroscopy analysis of the materials. Raman spectra of GR-CMF composite was taken using a Dong Woo 500i Raman spectrometer from Korea equipped with a charge-coupled detector. Amperometric *i-t* curve was taken using CHI1205B electrochemical work station with the laccase immobilized GR-CMF composite modified SPCE as a working electrode. Saturated Ag|AgCl and Pt wire were used as a reference and auxiliary electrodes for the electrochemical measurements. The electrochemically active surface area of the biosensor modified SPCE was 0.25 cm^2^, and was calculated using Randles–Sevcik equation^51^.

### Fabrication of laccase biosensor

Laccase (5 mg mL^−1^) stock solutions was freshly prepared using 0.1 M sodium phosphate buffer (pH 5.0) and stored at −4 °C when not in use. The GR-CMF composite was prepared by dispersing of GR (5 mg mL^−1^) into the CMF solution with the help of ultrasonication for 30 min. Meanwhile, the CMF solutions were prepared by the sonication of 10 mg mL^−1^ of CMF into the doubly distilled water for 45 min. Then, 6 μL (optimum, see Fig. 4 inset) of the as-prepared GR-CMF composite solution was drop coated on unmodified SPCE and dried at room temperature. For the biosensor fabrication, about 6 μL of laccase stock solution was dropped on as-prepared GR-CMF composite modified SPCE and dried at room temperature. The GR dispersions (5 mg mL^−1^) were prepared using DMF and HB immobilized GR and CMF modified SPCEs were prepared by drop coating of laccase (6 μL) on GR and CMF modified SPCEs. All electrochemical measurements were performed in a room temperature. The as-prepared laccase biosensor modified SPCE was stored at −4 °C under dry conditions when not in use.

## Additional Information

**How to cite this article:** Palanisamy, S. *et al*. A novel Laccase Biosensor based on Laccase immobilized Graphene-Cellulose Microfiber Composite modified Screen-Printed Carbon Electrode for Sensitive Determination of Catechol. *Sci. Rep.* **7**, 41214; doi: 10.1038/srep41214 (2017).

**Publisher's note:** Springer Nature remains neutral with regard to jurisdictional claims in published maps and institutional affiliations.

## Figures and Tables

**Figure 1:**
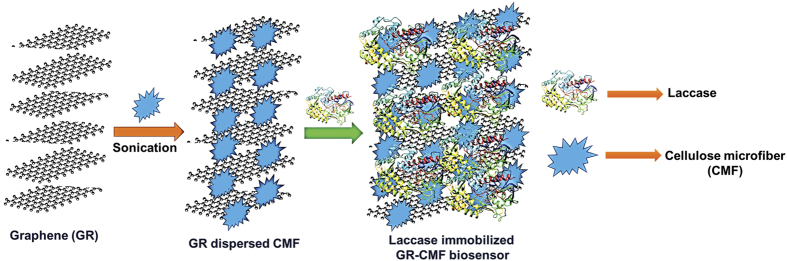
Schematic representation for the fabrication of laccase biosensor.

**Table 1:** Analytical comparison of the as-prepared GR-CMF/laccase modified SPCE with the previously reported laccase biosensors for determination of CC.

Biosensor	Detection limit (μM)	Linear range (μM)	Sensitivity (μA mM^–1^ cm^−2^)	Ref.
1RGO-PdCu NCs/Lac/GCE	1.52	up to 1155.0	12.65	42
2N-OMC/PVA/Lac/AuE	0.39	up to 8.98	0.29	43
3Cu-OMC/PVA/Lac/AuE	0.67	up to 15.75	0.104	44
4Cu/CNFs/Lac/Nafion/GCE	1.18	up to 9760.0	33.1	45
5Lac/AP-rGOs/Chit/GCE	7.0	up to 700.0	1.12	46
6Lac/MCNT-CS/GCE	0.66	up to 30.0	NR	47
7Lac/PANI/GCE	2.07	up to 19.36	0.7067	48
8Lac/GC-rGO/GCE	0.076	up to 15.0	0.0065	52
9Lac/MCNT/GCE	2.0	up to 1000.0	NR	49
10Lac/CS/ZnO/GCE	0.290	up to 100.0	0.001052	50
GR-CMF/laccase/SPCE	0.085	up to 209.7	0.932	This work

^1^Laccase immobilized on reduced graphene oxide supported palladium–copper alloyed nanocages.

^2^Laccase immobilized on nitrogen-doped ordered mesoporous/PVA matrix.

^3^Laccase immobilized in copper-containing ordered mesoporous carbon/chitosan matrix.

^4^Laccase immobilized on Electrospun copper/carbon composite nanofibers.

^5^Laccase immobilized onto 1-aminopyrene functionalized reduced graphene oxide.

^6^Laccase immobilized on carbon nanotubes–chitosan composite.

^7^Laccase immobilized on polyaniline.

^8^Laccase immobilized on reduced graphene oxide–glycol chitosan nanohybrid.

^9^Laccase immobilized on multi-walled carbon nanotubes.

^10^Laccase immobilized in a ZnO sol-gel with chitosan.

**Table 2:** Determination of CC in water samples using the GR-CMF/laccase modified SPCE.

Samples	Detected (μM)	Added (μM)	Found (μM)	Recovery (μM)	RSD (%)
Tap water	0	2.0	1.96	98.0	3.3
	1.96	2.0	3.98	99.5	3.1
River water	0	2.0	1.91	95.5	3.8
	1.91	2.0	3.81	97.4	4.4

The relative standard deviation (RSD) is relative to 5 measurements.
